# Simulating Food Web Dynamics along a Gradient: Quantifying Human Influence

**DOI:** 10.1371/journal.pone.0040280

**Published:** 2012-07-02

**Authors:** Ferenc Jordán, Nerta Gjata, Shu Mei, Catherine M. Yule

**Affiliations:** 1 The Microsoft Research – University of Trento Centre for Computational and Systems Biology, Trento, Italy; 2 School of Science, Monash University, Selangor DarulEhsan, Malaysia; Dalhousie University, Canada

## Abstract

Realistically parameterized and dynamically simulated food-webs are useful tool to explore the importance of the functional diversity of ecosystems, and in particular relations between the dynamics of species and the whole community. We present a stochastic dynamical food web simulation for the Kelian River (Borneo). The food web was constructed for six different locations, arrayed along a gradient of increasing human perturbation (mostly resulting from gold mining activities) along the river. Along the river, the relative importance of grazers, filterers and shredders decreases with increasing disturbance downstream, while predators become more dominant in governing eco-dynamics. Human activity led to increased turbidity and sedimentation which adversely impacts primary productivity. Since the main difference between the study sites was not the composition of the food webs (structure is quite similar) but the strengths of interactions and the abundance of the trophic groups, a dynamical simulation approach seemed to be useful to better explain human influence. In the pristine river (study site 1), when comparing a structural version of our model with the dynamical model we found that structurally central groups such as omnivores and carnivores were not the most important ones dynamically. Instead, primary consumers such as invertebrate grazers and shredders generated a greater dynamical response. Based on the dynamically most important groups, bottom-up control is replaced by the predominant top-down control regime as distance downstream and human disturbance increased. An important finding, potentially explaining the poor structure to dynamics relationship, is that indirect effects are at least as important as direct ones during the simulations. We suggest that our approach and this simulation framework could serve systems-based conservation efforts. Quantitative indicators on the relative importance of trophic groups and the mechanistic modeling of eco-dynamics could greatly contribute to understanding various aspects of functional diversity.

## Introduction

Systems approaches to exploring the role of functional diversity in communities may help in quantifying the roles species play and their relative importance. Exploring the role of functional diversity may be especially important in tropical ecosystems where many species are rare, which makes the entire community more difficult to study and the importance of individual species more challenging. Despite being under-represented in the literature, the functioning of freshwater tropical ecosystems is amongst the most heavily impacted by human disturbance [1].

One way to evaluate individual species in a systems context is to study the food web of the community [2]. Such structural analyses are relatively fast and easy but the utility of structural food webs in capturing important information about functions and processes is often questioned. Dynamical models in contrast provide essential information especially if one needs to understand changes in interaction strength and abundances, with the structure of the food web being almost constant. In this case, understanding the dynamical behavior of the major components of the ecosystem and quantifying their sensitivity to different conditions can provide a truly functional view of their diversity and redundancy.

In this paper, we present a stochastic dynamical simulation and sensitivity analysis of a food web of the Kelian River, Borneo. The Kelian River has been severely impacted by sediment pollution due to gold mining activities which have caused increasing disturbance with increasing distance downstream [3]. Since the 1950s, alluvial miners searched for gold along the Kelian and its tributaries, and Indonesia' second largest open pit gold mine operated beside the river from 1991 until 2005. Based on a massive field data collection (16,424 macroinvertebrates from at least 179 species collected during 1990, 1993, 1994 and 1995) [3], [4] plus studies on the fish communities at the same sites, we build a dynamical food web model at six sites along the river representing different levels of human disturbance. We first analyse the structural properties and the simulated dynamics of the pristine river ecosystem, then we compare the most important results to the ones derived for the other five sites. In particular, we use the simulated behavior of shredders as a key indicator group (following [5] but see [6]). Shredders break down leaf litter in streams and make it available to other aquatic fauna, and thus they are a vital link between aquatic and terrestrial ecosystems [5], and they can be sentinels of environmental perturbations [5].

## Methods

### The study area

The Kelian River arises in pristine rainforest near the centre of Borneo. Six sites were sampled along the river with site 1 located in pristine rainforest and site 2 next to the open cut mine (aquatic invertebrate sampling was permitted under the terms and conditions applying to the Indonesian contract of work for PT Kelian Equatorial Mining). The impact of alluvial mining was evident from just below site 1, above the mine, escalating in severity with increasing distance downstream. Organic pollution due to the disposal of human waste into the river also had a negative effect on the river fauna at the downstream sites [4].The substrate characteristics of riffle and pool habitats and the size of the river were similar at all sites, suggesting that discharge did not vary greatly among sites. The width varied from ∼15 to 25 m, and the maximum depth in the riffles was ∼30–40 cm at all sites. The climate is tropical with an average annual rainfall of 4000 mm. Average daily flow of the Kelian River is ∼10 m^3^ s^−1^, but the mean daily flow can vary greatly: measurements taken near site 3 ranged from 26.5 m^3^ s^−1^ in April down to 3.0 m^3^ s^−1^ in October 1994.

Based on earlier measurements, a number of abiotic indicators are also available for these sites [3]. Here we used temperature and turbidity data, as these are among the easiest ones to interpret biologically. Decreases in macro-invertebrate richness and abundance were significantly correlated with elevated turbidity [3].

### Food web construction

The fauna and flora of the river has been extensively sampled at six study sites. Samples collected in different years−September 1990 (wet season), August 1993 (dry season), June 1994 (dry season) and March 1995 (wet season)−provided quite consistent results, with no significant variation in faunal abundance or richness indicating no differences in taxonomic composition among years (3) or seasons. Thus, we aggregated data across years and seasons to assemble the food webs.

Unlike temporal aggregation, spatial aggregation did not make sense because the different segments of the river differ in terms of their fauna, the natural environmental conditions, and human effects. Taxonomic aggregation is always a key issue in food web research. Aggregating species-level information into larger functional groups (trophic groups) is not easy to standardize but typically increases the functionality and reality of the study. Our networks are composed of 12<N<15 graph nodes each representing a trophic group. We emphasize that these low-resolution food webs are not worse, only different from the large-resolution food webs [7]. Studying “small” (i.e. low-resolution) food webs provides different results [8] and functional diversity must also be understood at this level of resolution as well because in some cases we do not face the extinction of individual species but of whole functional groups [9].

**Figure 1:**
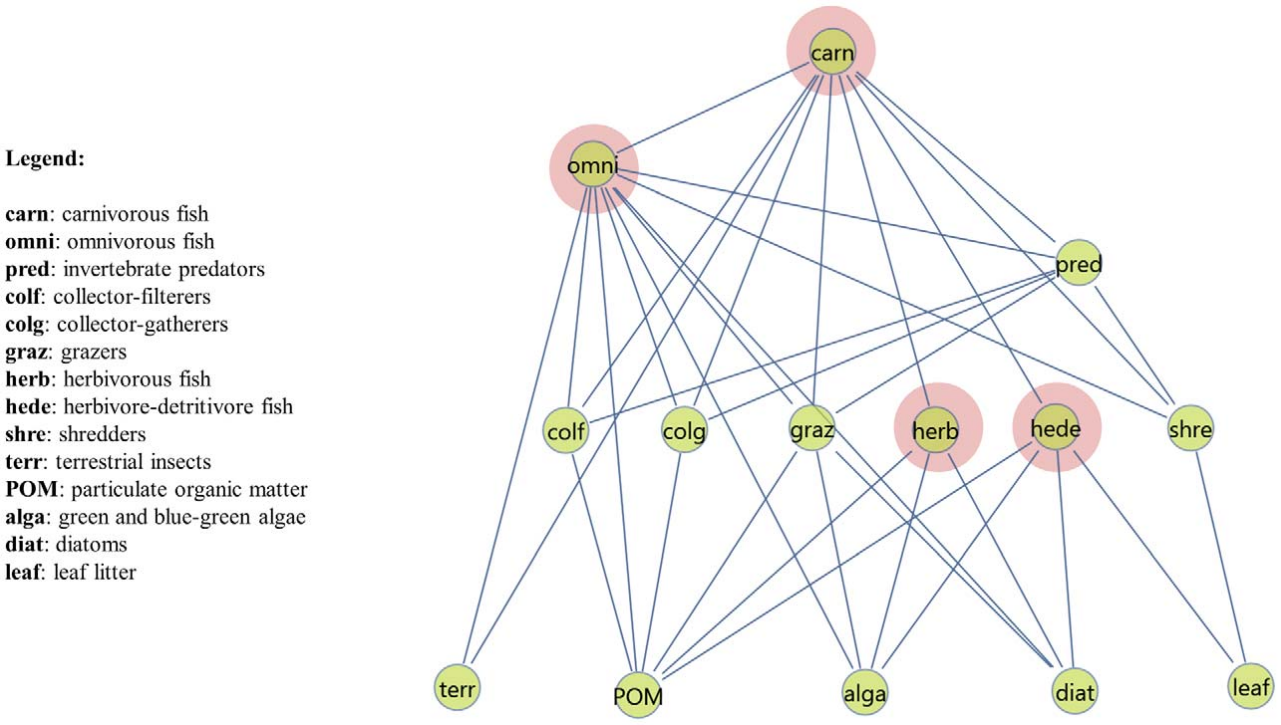
The food web of Kelian River at sampling site 1 (the pristine river segment). The inset on the left provides the full names of trophic groups. Nodes with pink background represent fish groups. There are two symmetrical links (PRED/OMNI and CARN/PRED), otherwise the network would be acyclic. Network drawn by COSBILAB Graph [17].

The food web representing the trophic groups of the river ecosystem, is shown in Figure 1. At the bottom of the food web, two non-living (LEAF: leaf litter, POM: settled and suspended, coarse and fine particulate organic matter) and three living (TERR: terrestrial insects, DIAT: diatoms, ALGA: green and blue-green algae) components provide food. Six herbivorous groups consume the producers (COLF: invertebrate collector-filterers, COLG: invertebrate collector-gatherers, SHRE: invertebrate shredders, HEDE: herbivore-detritivore fish, HERB: herbivorous fish, GRAZ: invertebrate grazers), and these are consumed by omnivores (OMNI: omnivorous fish) and higher predators (PRED: invertebrate predators, CARN: carnivorous fish). In downstream river segments, two additional trophic groups appear: HUMW (human waste) and FILA (filamentous bacteria): these are producer groups in the system. For a detailed taxonomic description, see [3], [4].

### Spatial variability

There are marked differences in the composition of the river community in the six sites. Human waste (HUMW) appears only in the most human-influenced segment next to a village where the village toilet is a cubicle in the river (site 6). Conversely leaf litter (LEAF) is present everywhere except for this site where what little occurs is smothered by sediment deposits. Filamentous bacteria (FILA) are not present in the two most pristine sites, only from sites 3–6. The filamentous bacteria metabolise manganese in the water and deposit it on and in their mucilaginous secretions, forming a slimy coating on the rocks which traps sediment and organic detritus. Collector-filterers (COLF), grazers (GRAZ) and shredders (SHRE) are the groups most sensitive to human influence, and they are missing at sites 4 and 6 although it should be noted that some of these species changed their diets, becoming collector-gatherers at these polluted sites. The remaining components (ALGA, CARN, COLG, DIAT, HEDE, HERB, OMNI, POM, PRED and TERR) were collected all along the river. The number of trophic groups in the food web were 14, 14, 15, 12, 15 and 12 from site 1 to site 6.

### Structural analysis

The food web of the pristine river is characterized by four topological network metrics as follows:

### Node degree

The most local index about the topology of a network is the *degree* of a node (*D*). This is the number of other nodes connected directly to it. In a food web, the degree of a node *i* (*D_i_*) is the sum of its prey (in-degree, *D_in,i_*) and predators (out-degree, *D_out,i_*):
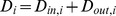
Degree provides information about local connectedness, and highly connected nodes are also called “hubs”. In network analysis, degree can be used as a reference index, providing a minimal characterisation of node position.

### Keystone indices

Nodal degree considers only the links directly connected to a node. In this section we describe indices that consider information in addition to such direct neighbours. The keystone index (*K*) [10] derives predominantly from the pioneering application of the “net status” index [11]. The keystone index of a species *i* (*K_i_*) is defined as:
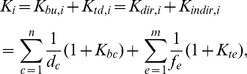
Where *n* is the number of predators eating species *i*, *d_c_* is the number of prey of its *c* ^th^ predator and *K_bc_* is the bottom-up keystone index of the *c* ^th^ predator. And symmetrically, *m* is the number of prey eaten by species *i*, *f_e_* is the number of predators of its *e* ^th^ prey and *K_te_* is the top-down keystone index of the *e* ^th^ prey. For node *i*, the first sum in Eq. (2) (*i.e.*∑1/*d_c_*(1+*K_bc_*)) quantifies the bottom-up effect (*K_bu,i_*) while the second sum (*i.e.*∑1/*f_e_*(1+*K_te_*)) quantifies the top-down effect (*K_td,i_*). After rearranging Eq (2), terms including *K_bc_* and *K_te_* (*i.e.*∑*K_bc_*/*d_c_*+∑*K_te_*/*f_e_*) refer to indirect effects for node *i* (*K_indir,i_*), while terms not containing *K_bc_* and *K_te_* (*i.e.*∑1/*d_c_*+∑1/*f_e_*) refer to direct ones (*K_dir,i_*). Both *K_bu,i_*+*K_td,i_* and *K_indir,i_*+*K_dir,i_* equals *K_i_*. The degree of a node in a network (*D*) characterises only the number of its connected (neighbour) points, while the keystone index gives information also on how these neighbours are connected to *their neighbours*. It emphasises vertical over horizontal interactions (*e.g.* trophic cascades as opposed to apparent competition) but characterises positional importance by separating indirect from direct, as well as bottom-up from top-down effects in food webs. This index may be used for analysing a network especially if top-down and/or bottom-up processes seem to be of particular interest. This can be the case of pollution, eutrophication or overfishing.

### Betweenness centrality

A measure of positional importance quantifies how frequently a node *i* is on the shortest path between every pair of nodes *j* and *k*. This index is called “betweenness centrality” (*BC*) and used routinely in social network analysis [12]. The standardised index for a node *i* (*BC_i_*) is:
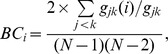
where *i*≠*j* and *k*. *g_jk_* is the number of equally shortest paths between nodes *j* and *k*, and *g_jk_(i)* is the number of these shortest paths to which node *i* is incident (of course, *g_jk_* may equal one). The denominator is twice the number of pairs of nodes without node *i*. This index thus measures how central a node is, in the sense of being incident to many of the shortest paths in the network. If *BC_i_* is large for trophic group *i*, we can expect larger areas of the network to become separated, because nodes of high betweenness typically connect them, making network communication (information flow) faster or shorter. A typical situation for a high BC value is the position of sardine or anchovy in marine wasp-waist food webs.

### Topological importance

In a network with undirected links, indirect effects can spread in any directions. Considering only indirect chain effects [13]–[15] and a binary (unweighted) network, we define *a_n,ij_* as the effect of *j* on *i* when *i* can be reached from *j* in *n* steps. The simplest mode of calculating *a_n,ij_* is when *n* = 1 (*i.e.* the effect of *j* on *i* in 1 step): *a*_1,*ij*_ = 1/*D_i_*, where *D_i_* is the degree of node *i* (*i.e.* the number of its direct neighbours including both prey or predatory species). The idea here is that any neighbour of node *i* will influence it more if node *i* has fewer neighbours.

We assume that indirect chain effects are multiplicative and additive. For instance, we wish to determine the effect of *j* on *i* in 2 steps, and there are two such 2-step pathways from *j* to *i*: one is through *k* and the other is through *h*. The effects of *j* on *i* through *k* is defined as the product of two direct effects (*i.e.a*_1,*kj*_×*a*_1,*ik*_), therefore it is termed multiplicative. Similarly, the effect of *j* on *i* through *h* equals to *a*_1,*hj,*1_×*a*_1,*ih*_. To determine the 2-step effect of *j* on *i* (*a*_2,*ij*_), we simply sum up those two individual 2-step effects (*i.e.a*_2,*ij*_ = *a*_1,*kj*_×*a*_1,*ik*_+*a*_1,*hj*_×*a*_1,*ih*_) and therefore this is termed additive.

When the effect of step *n* is considered, we define the effect received by species *i* from all species in the same network as:
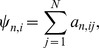
which is equal to 1 (*i.e.* each species is affected by the same unit effect.). Furthermore, we define the *n*-step effect originated from a species *i* as:
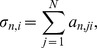
which may vary among different species (*i.e.* effects originated from different species maybe different). Here, we define the topological importance of species *i* when effects “up to” *n* steps are considered as:
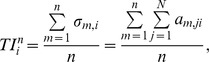
which is simply the sum of effects originating from species *i* up to *n* steps (one plus two plus three…up to *n*) averaged by the maximum number of steps considered (*i.e. n*). This index was originally introduced for studying host-parasitoid networks [16]. All of these four indices provide some information about the centrality of nodes in the network, as a proxy for functional importance. All metrics were calculated by the COSBILab Graph software [17]. For more detailed descriptions of indices, see [18].

Apart from characterizing the position of individual nodes, the *TI* index also provides a measurement for interaction strength. Between each *ij* ordered pair of nodes, the topological constraint on the strength of the interaction was calculated (their structural connectedness). In the rank of these *ij* interaction strengths, one can study the ranks of certain types of effects. We analyzed four kinds of effects: 35 prey-predator and 35 predator-prey interactions, 9 trophic cascades and 14 self-loop interactions (the latter being the main diagonal of the interaction matrix). In the *n* = 14 interaction matrix, the *n* ^2^ = 196 *ij* effects included also 103 non-classified indirect interactions.

### Dynamics

Some of the trophic groups in our system contain only a few individuals (e.g. CARN). In this case, stochasticity causes a fair amount of internal noise in systems dynamics. It is less likely that certain processes will follow simple ecological laws (although the behavior of more abundant groups can be more deterministic). For this reason, we use an individual-based, stochastic framework for modeling the dynamics of the ecosystem.

With the development of computer science, there are novel tools and approaches to address the complex problems emerging in computational ecology [19]–[21]. Some of these tools make it possible to simulate the behavior of complex ecological systems characterized by a number of parameters [22], [23]. For the dynamical analysis, we constructed six individual-based food web models and simulated their behavior by a stochastic simulator using the Gillespie algorithm [24]. The model was constructed in the BlenX framework [25], [26] and analyzed by its stochastic simulator module that enables computationally cheap and realistic food web simulations [23], [27] (see Appendix S1).

For parameterizing the dynamical model, we needed values internal (e.g. death rate) and external (e.g. predation rate) to the trophic groups. Based on field samplings [4], the number of individuals was approximated for each trophic group. In the case of some groups it was relatively easy, based on the prey item data. For ALGA and DIAT it was much harder, so we approximated some realistic number of individuals. Interaction rates were inferred from prey preference data [4]. For the dynamical model, we needed birth rate and death rate parameters as well. Lacking these data, we used hypothetical values for parameterizing all networks. Actual parameter sets needed to be slightly refined in a way that let the model behave in a quasi-balanced way (no mass extinctions and exponential growths). For example, COLF interaction rate was fine-tuned from 0.05 to 0.00014. However, most of these minor adjustments concerned the hypothetical parameters, not the measured ones. In such a complex model with so many parameters, it is clear that most of the values are at best approximations (see all parameters for site 1 in Appendix S2).

All reference simulation runs were sampled at time *t*. At time *t*, the mean and the standard deviation of all components were registered, based on *s* simulations. We note that the mean of many stochastic simulations approaches the results obtained from deterministic simulations, but for a small *s* number of simulations, the variability of dynamical behavior is also informative.

The basic (reference) model was then subject to sensitivity analysis. For estimating the effect of species i on the mean population size of species j, we first define the reference value of population density for species j (A_j_) in the absence of any disturbance
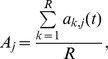
where *R* simulations are performed and, for each run *k*, the population size of species *j* in the undisturbed system (a_k,j_) is recorded at time *t*. The initial number of individuals for each component was then halved, one by one, and the mean values of all components were recorded after time *t*, as before, for the same *R* number of simulations for each disturbed parameter. The value of population density for species *j*, after disturbing species *i* is
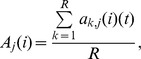
and the relative response of species *j* to disturbing species *i* is
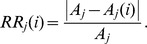
The relative response is normalized over all the living groups (n):
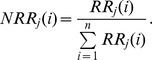
The community importance if species *i* equals
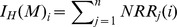
and, symmetrically, if the standard deviation is considered instead of the mean of the R simulations, we provide a community importance metric quantifying the effects on variability of the population dynamics of other groups:
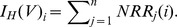
These normalized relative response metrics (in mean and variation) measure the sensitivity of the system to disturbing component *i* (*I_H_*, where H stands for the Hurlbert response function [28]). These simulation-based values are dynamical measurements of community importance.

Inspired by the classical concept of keystone species [29], [30], we expressed both dynamical importance metrics in a way considering the population size of the trophic group. *K_H_(M)* = *I_H_(M)*/*ps* and *K_H_(V)* = *I_H_(V)*/*ps*, where *ps* is the number of individuals in the trophic group. However the original approach suggested using biomass, we believe that the number of individuals is equally good if not better, given our individual-based model. All importance indices used are summarized in Table 1.

**Table 1:** The structural and dynamical importance indices used in this paper (see definitions and explanations in text).

Network indices				
*name*	*symbol*	*type*	*brief description*	*reference*
degree	D	structural	number of neighbours	[12]
keystone index	K	structural	vertical interactions, also indirect	[10]
betweenness	BC	structural	appearing in shortest paths	[12]
topological importance	TI	structural	neighbourhood in all directions, also indirect	[16]
community importance	I_H_(M)	dynamical	effects on mean population size of others	[23]
community importance	I_H_(V)	dynamical	effects on population size variability of others	[23]
per capita community importance	K_H_(M)	dynamical	effects on mean population size of others (per capita)	here
per capita community importance	K_H_(V)	dynamical	effects on population size variability of others (per capita)	here

## Results

### The pristine river ecosystem

**Figure 2:**
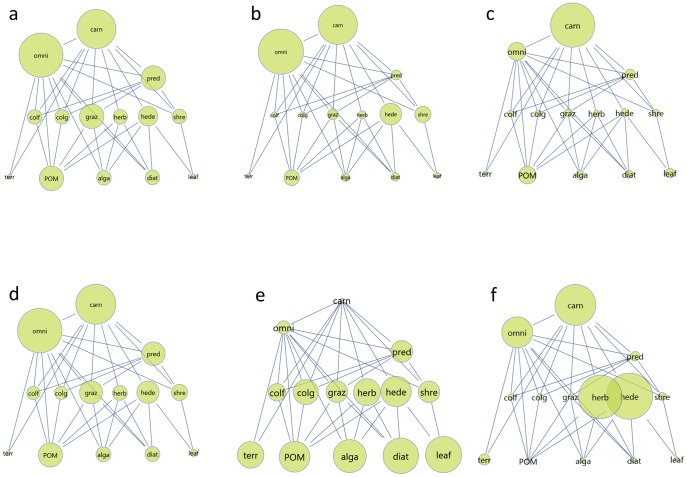
The food web of the pristine river (site 1), size of nodes is proportional to six measures of importance: *D* (a), *nBC* (b), *K* (c), *TI^3^* (d), *I_H_(V)* (e) and *K_H_(V)* (f). Network drawn by COSBILAB Graph [17].

In site 1, all trophic groups occur apart from FILA and HUMW. In the food web of 14 nodes, OMNI and CARN are clearly the most connected nodes, three indices (*D*, *nBC*, *TI^3^*) supporting more OMNI and the *K* index emphasizing the role of CARN (see Figures 2a–d and Table 2). Based on the rankings of these indices, OMNI and CARN are really of outstanding importance. Some other groups are of highly variable centrality, according to these four indices. For example, the rank of SHRE ranges from 4 (suggested by *nBC*) to 10 (suggested by *K*; because of the ties, its rank is 9.5 according to *D*). Terrestrial insects (TERR) are clearly the structurally least important group.

**Table 2:** Importance ranks of trophic groups in the food web of the pristine river (site 1).

	D		nBC		K		TI^3^		I_H_(M)		I_H_(V)		K_H_(M)		K_H_(V)
omni	10	omni	22,811	carn	13	omni	2,07	graz	0,0818	leaf	0,0915	carn	0,0144	hede	0,0081
carn	9	carn	19,426	omni	4,53	carn	1,88	alga	0,0780	diat	0,0903	herb	0,0071	herb	0,0076
graz	6	hede	9,515	POM	4,37	POM	1,19	diat	0,0765	alga	0,0860	omni	0,0057	carn	0,0071
POM	6	shre	6,404	pred	2,07	pred	1,16	colf	0,0751	POM	0,0821	hede	0,0057	omni	0,0052
pred	6	POM	6,081	leaf	1,82	graz	1,14	pred	0,0745	hede	0,0813	terr	0,0014	terr	0,0014
hede	5	graz	3,419	alga	1,29	hede	1,11	colg	0,0741	herb	0,0755	pred	0,0012	pred	0,0011
alga	4	pred	2,94	diat	1,29	shre	0,85	terr	0,0736	terr	0,0748	shre	0,0008	shre	0,0008
colf	4	alga	2,042	hede	1,28	alga	0,79	shre	0,0736	colg	0,0723	colf	0,0004	colf	0,0003
colg	4	diat	2,042	graz	1,21	diat	0,79	carn	0,0722	pred	0,0666	colg	0,0002	colg	0,0002
diat	4	herb	1,816	shre	1,04	herb	0,79	herb	0,0707	shre	0,0663	alga	0,0002	diat	0,0002
herb	4	leaf	0,641	herb	0,78	colf	0,72	POM	0,0687	graz	0,0661	diat	0,0002	alga	0,0002
shre	4	colf	0,534	colf	0,71	colg	0,72	leaf	0,0673	colf	0,0593	graz	0,0001	leaf	0,0001
leaf	2	colg	0,534	colg	0,71	leaf	0,45	omni	0,0572	omni	0,0524	POM	0,0001	POM	0,0001
terr	2	terr	0	terr	0,23	terr	0,35	hede	0,0566	carn	0,0355	leaf	0,0001	graz	0,0001

Ranking is based on topological (*D*, *nBC*, *K*, *TI^3^*) and dynamical (*I_H_(M)*, *I_H_(V)*) measures, as well as keystone indices considering also population size (*K_H_(M)*, *K_H_(V)*). The *I_H_(M)* and *I_H_(V)* indices for all groups in each of the six sites are given in Appendix S5.

**Figure 3:**
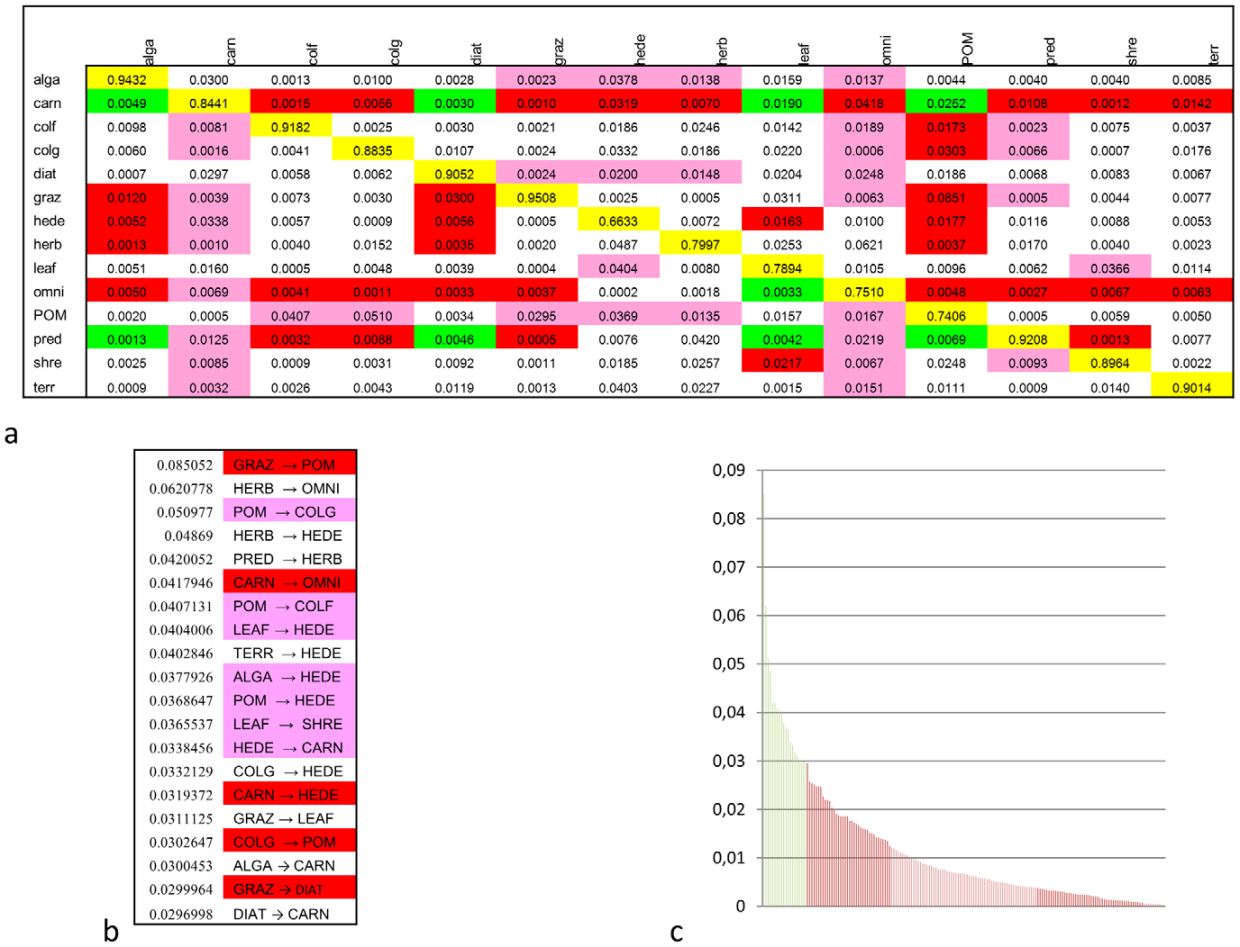
The matrix of simulated dynamical interaction strengths in site 1(from groups in row *i* to groups in column *j*). Four simple interaction types were analyzed: indirect self-loop is yellow, predator-prey interaction is red, prey-predator interaction is purple and trophic cascade is green (a). None of these interactions is in white. In (b), the 20 strongest simulated interactions are shown (apart of the 14 self-loops that are obviously the strongest ones). Direct (red and purple) and indirect (only white, as no green trophic cascade appears in the top 20) interactions are well-mixed. The strongest one is a direct effect from GRAZ to POM, the second strongest effect is indirect (from HERB to OMNI). The shown 20 interactions are followed by 162 weaker effects, their rank is shown in (c). Here, the *x* axis is the rank of interactions, while the *y* axis is their strength shown in (a).

The food web contains 35 trophic links, so the 196 (14*14) effects of the interaction structure can be classified as 14 self-loops, 35 predator-prey effects, 35 prey-predator effects and 9 trophic cascades. In the case of A→B trophic cascades, 23 different A-x-B pathways exist between 9 different A–B pairs of groups (cascades where A directly feeds on B have been classified as predator-prey effects). The remaining 103 effects do not belong to any of the above four types of interactions. Figure 3a shows this classification of effects.

Dynamical simulations and sensitivity analysis suggest that grazers (GRAZ) are the group with the largest community effect (*I_H_(M)*). Disturbing the structurally most important groups (OMNI, CARN) generates much smaller community response (see Figure 2e, Table 2 and Appendix S3). However, the differences between the relative importance of the groups are smaller here than based on the topological indices.

If dynamical importance (*I_H_(M)*) is divided by the number of individuals (*ps*), the resulting keystone index (*K_H_(M)*) suggests CARN to be the keystone group, also OMNI with somewhat less importance and still with HERB in second place (see Table 2). Here, GRAZ is much less important than according to any of the previous indices. Figure 2f shows the outstanding keystone role of CARN in the system.

An analysis at the level of individual interactions shows that indirect effects are roughly as important as direct ones in governing ecosystem dynamics. Figure 3b provides the rank of the strongest 20 simulated effects: direct (predator-prey in red and prey-predator in purple) and indirect (white) effects are well-mixed in the rank. The shown 20 effects contain some outstandingly strong indirect effects as well (see Figure 3c). This indirect determination [31] can be one reason why structure is poor in predicting dynamics (even if indirect structural indices have also been used).

An additional measure of dynamical importance considers the effect on the dynamical variability of other groups, instead of the absolute change in their population size (*I_H_(V)*). In some cases, the variability of population dynamics maybe more important than the actual size of the population. For example, because variability is a proxy for adaptability, this is true not only in a genetic, but also in a population dynamical sense (it is easier to find optimal evolutionary ecological strategies with experiencing different population sizes). Also, in the case of small populations being at the brink of extinction, a more variable behavior may drive (drift) the population to extinction, while a smaller but less wildly behaving population can be safer. It has been suggested recently [32] that considering variability explicitly could be of high importance for conservation studies. Our *I_H_(V)* index quantifies community importance based on the influence on dynamical variability. It suggests that disturbing the producers (LEAF, DIAT, ALGA, POM) will generate the most variable behavior for other groups (see Figure 2e and Table 2). The distribution is quite uniform, just like in the case of the *I_H_(M)* index. Transforming also the *I_H_(V)* index to a *K_H_(V)* keystone index provides results very similar to those based on *K_H_(M)* (but indicating the highest role for HEDE) and prioritizing the same four groups as keystones (see Table 2).

### Food web variation along the river

Some trophic groups are missing in some locations along the river. For example invertebrate filterers, grazers and shredders were eliminated due to pollution at sites 4 and 6. We simulated and analyzed the food webs of the six study sites only from a dynamical point of view, providing topological results only for site 1 (see above). Beyond the composition of trophic groups and the consequent structural changes of the network, several dynamical parameters also differ between different sites. Dynamical simulations and sensitivity analysis quantify the functional effects of these changes.

Based on *I_H_(M)*, invertebrate grazers (GRAZ) keep their leading position in sites 2 and 3 but downstream they are either missing (sites 4 and 6) or ranked lower in importance (site 5). Invertebrate predators (PRED) are of average importance in the pristine site and of low importance in the middle of the river but the most human-influenced ecosystem is most sensitive to them (in site 6). Also, the role of filamentous bacteria (FILA) is very important when they are present (only downstream, indicating pollution). Invertebrate shredders (SHRE) gradually lose their importance and finally disappear from the system (lacking at sites 4 and 6). However, their abundance has been shown to decrease from higher to lower elevations in tropical streams in Peninsular Malaysia [33]. The community importance series of each group is shown in Appendix S4 (see Appendix S5 for the numerical values).

**Figure 4:**
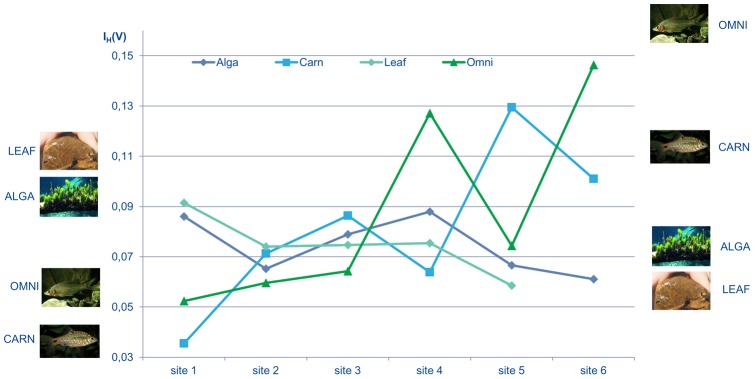
The *I_H_(V)* value for four selected trophic groups at each site. The top-down effects of fish on the dynamical variability of other groups in increasing in the more human-influenced site.

Using the community importance measure focusing on dynamical variability (*I_H_(V)*), the dominant role of producers (LEAF, DIAT, ALGA, POM) is decreasing downstream, especially in the most human-influenced sites (site 2 and 6). At site 6, LEAF disappears (see Appendix S5). Grazers (GRAZ) are not important, according to this measure, at any sites. The group that is becoming more consistently important is the omnivores (OMNI). Their importance is greatest at site 6, so the dynamical variability of the human-dominated river ecosystem is mostly sensitive to changes of the omnivorous community (Figure 4, see also Appendix S4). In general, it can be seen in Figure 4 that community sensitivity in terms of dynamical variability is increasing from the pristine and quasi-natural locations towards increasing human influence. Based on the dynamically most important groups, we can say that bottom-up control is replaced by the predominant top-down control regime.

**Figure 5:**
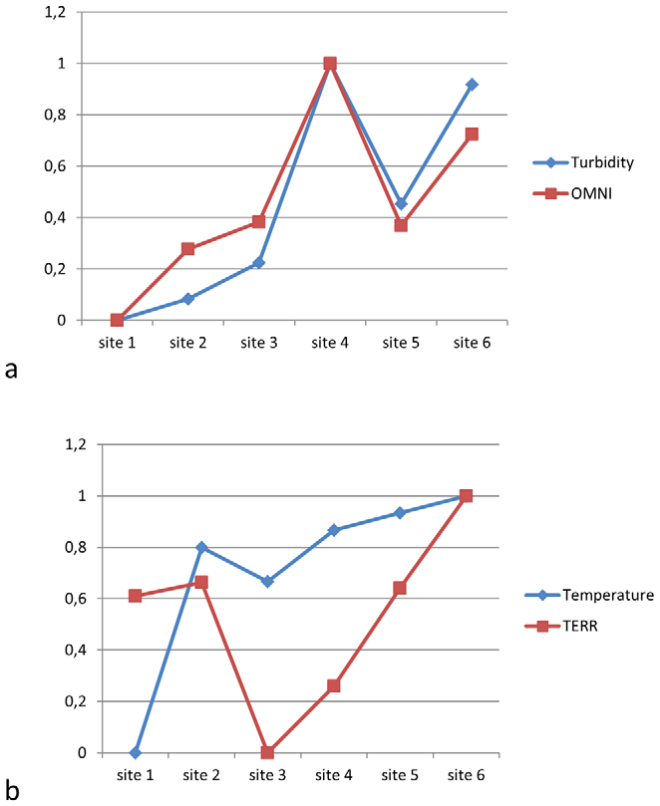
Abiotic variables (turbidity in (a) and temperature in (b)) followed by biotic ones (*I_H_(M)* for OMNI in (a) and TERR in (b)). To help visual comparability, values have been transformed in such a way that the minimum and the maximum value be equal to 0 and 1, respectively. The *x* axis is the series of study sites.

Some of the above tendencies show significant correlation with abiotic indicators of the river ecosystem [3]. Figure 5a shows the spatial variation of turbidity and the dynamical importance of OMNI (*I_H_(M)*), respectively, along the river. In turbid waters with higher amounts of suspended solids OMNI plays a larger role in ecosystem dynamics. Figure 5b shows the series of temperature and the importance of terrestrial insects (TERR), respectively. The sign of temperature changes is always mirrored by the changing importance of terrestrial insects.

## Discussion

We performed stochastic food web simulations and sensitivity analysis on six food web models assembled along a gradient of human influence in Kelian River, Borneo.

### Comparison of the structural versus dynamical results

Beyond structural analysis, we quantified the relative importance of trophic groups using dynamical simulations. We studied a highly aggregated system containing trophic groups of organisms. We believe that, even if biodiversity is typically studied at the level of species, the functional diversity of ecosystems should also be studied (or maybe more) at the level of functional groups. Structural results suggest that the key groups in the pristine river ecosystem are at higher trophic levels, while the disturbance of producers generated stronger community response during the simulations. Thus, we have found that network structure only very poorly predicts network dynamics in this study site of the system. Also, several measures provide complementary information and a multidimensional evaluation of the system may help to better indicate and quantify the key components and processes. An important finding, potentially explaining the poor structure to dynamics relationship, is that indirect effects are at least as important as direct ones during the simulations. Most indices provided a keystone-like distribution of importance values calculated for the different groups [34]. Comparing the food webs described at different sites is interesting especially because of this contrast.

### Discussion of how food-web structure changes downstream

Our results suggest that the group of invertebrate shredders (SHRE) is one of the clearest indicators of human influence. This can be of particular interest, as shredders play an important role in food web development [35], breaking down leaves into smaller particles for consumption by other organisms such as collector gatherers and filterers. With increasing distance downstream, there tends to be less riparian vegetation providing an input of leaf litter, and furthermore the leaves tend to be smothered by sediment in the river. Algae and diatoms decrease downstream due to turbidity and sediment deposition impeding photosynthesis. Thus the key role of grazers (GRAZ) and different groups of producers is gradually replaced by other groups that better tolerate human influence (e.g. pollution, turbidity). For example, omnivorous (OMNI) and carnivorous fish (CARN) become increasingly important even if their abundances do not change. Omnivorous fish can tolerate changes in primary productivity caused by pollution, while the carnivores are not dependent on primary production. We did not find a strong cascading effect from carnivorous fish (CARN) down to algae (ALGA), despite the generally acknowledged role of trophic cascades in aquatic systems [36] but this is not surprising since ecosystem functioning in the Kelian River is dominated by the effects of pollution. It is imperative to emphasize the dominant roles of invertebrates in shaping ecosystem dynamics at several locations (see [37]). The finding that terrestrial insects (TERR) were of little importance yet their behavior shows some correlation with temperature may be interesting for better understanding climatic effects on food web structure [38]. Terrestrial insects typically fall in from overhanging riparian vegetation, so less vegetation results in both fewer terrestrial insects and also less shading leading to higher temperatures.

Our approach is based on the river continuum concept (evaluating different roles of particular trophic groups in different segments of the river) and a systems perspective on ecology and conservation biology (providing holistic, quantitative indicators for the relative importance of groups). The presented indices of dynamical community importance may help setting conservation priorities, managing rare species and better understanding ecosystem fragility. We propose that the approach presented in this paper may contribute to the framework of systems-based, quantitative conservation biology [39].

## Supporting Information

Appendix S1 A brief introduction to the BlenX process algebra.
(DOC)

Appendix S2 The parameters used for the simulation of the food web at site 1.
(DOC)

Appendix S3 The food web at site 1, size of nodes being proportional to *I_H_(M)* and *K_H_(M)*.
(DOC)

Appendix S4 The *I_H_(M)* and *I_H_(V)* values of trophic groups at the six sites.
(DOC)

Appendix S5 The importance rank of trophic groups based on *I_H_(M)* and *I_H_(V)*.
(DOC)
