# Supplementary material for: Simulating Food Web Dynamics along a Gradient: Quantifying Human Influence
Source: PLoS One. 2012 Jul 2;7(7):e40280. doi: 10.1371/journal.pone.0040280 (PMC3388060; doi:10.1371/journal.pone.0040280)
Supplement: Appendix S1 — A brief introduction to the BlenX process algebra. (DOC) [file pone.0040280.s001.doc]

**Appendix S1:**

We model the dynamics of this hierarchical system in a stochastic framework, written in the process algebra-based BlenX programming language (Dematté et al., 2008). BlenX is a stochastic programming language explicitly designed to model interactions of biological entities. BlenX represents a biological entity as a box, composed by a set of interfaces and a process. Interfaces have associated types and represent the interaction capabilities of a box (e.g. predation). The internal program (P) codifies for the mechanism of the transformation of an interaction into an internal change (e.g. reproduction). A box can interact with other boxes by communication, using events. All the possible interactions are associated with a rate, expressing their dynamics. These rates are used when analyzing the system in order to derive the actual rate in stochastic simulation (Gillespie 1977).

A BlenX program is made up of (1) a program file for the definition of the main entities and their possible interactions, (2) an interfaces file for the quantitative information about the system and (3) an optional declaration file for the user-defined variables and functions. At the beginning of the program file, simulation time is specified. The program file contains the definition of all the boxes in the model: it ends with the initialization of the system using the keyword run specifying the initial population of a set of defined boxes. Boxes are defined using the keyword *bproc*. The general definition of a bio-process *B* is given by:

B : bproc = B [P]

where *B* is a non-empty list of interfaces and *P* is the internal process. Each interface site in the list *B* can be in one of the forms:

#(x;Δ;r) │ #h(x;Δ;r) │ #c(x;Δ;r)

where *x* is the subject, Δ is the type and *r* is the rate for the actions involving the interface inside the box. All the subjects and types must be different. The parameter *r* is optional; when not expressed, we consider a pre-defined *BASERATE* value.

Concerning the processes, these are defined either in the definition of the box or using the keyword *pproc*, starting from the following elementary processes:

x(y)? │ x(y)! │delay(r; x) │die(r) │ch(r; x;Δ) │ nil

The input action (x(y)?) intuitively means that a process is willing to receive on the channel *x* a

name that will replace the target variable *y*. The output action (x(y)!) sends a name *y* along a channel *x*. The change action (ch) changes the type of an interface with subject *x* into Δ. The “die” action eliminates the box.

Concerning communication, the rate is the one associated with the subject in the interface or in the *BASERATE* for the communication inside the box (intra-communication) and the one reported in the declaration file for the communication between two boxes (inter-communication, see below). The basic actions can be combined by sequential composition (symbol ”.”), parallel composition (symbol ”│”), choice (symbol ”+”), replication (keyword “rep”) or a conditional statement (”if - then”). The choice operator ”+” composes processes that can be alternatively executed. The parallel operator│ composes processes that execute concurrently and allows processes to communicate or synchronize when they perform complementary input/output actions on the same channel. The “rep” operator permits the definition of repetitive (recursive) and possibly infinite behaviors by producing as many copies in parallel as needed of the “rep” argument. Note that two boxes belong to the same species only if they are structurally equivalent.

A BlenX program can be executed within the Beta Workbench (BWB, Dematté et al. 2008), a set of tools to design, simulate and analyze models written in BlenX. The BWB simulator implements an efficient variant of the Gillespie algorithm (Gillespie 1977), allowing for using the quantitative information of BlenX to run stochastic simulations.
