# Supplementary material for: Simulating Food Web Dynamics along a Gradient: Quantifying Human Influence
Source: PLoS One. 2012 Jul 2;7(7):e40280. doi: 10.1371/journal.pone.0040280 (PMC3388060; doi:10.1371/journal.pone.0040280)
Supplement: Appendix S2 — The parameters used for the simulation of the food web at site 1. (DOC) [file pone.0040280.s002.doc]

**Appendix S2:**

The parameters used for the simulation of the food web at site 1. The left panel shows the parameters for trophic groups (init: initial number of individuals; death: death rate; birth: birth rate; when: primary production of producers) and the right panel shows the interaction rates (i: prey; j: predator). The latter defines food web topology. Parameters in red are measured in the field (and differ between the sites), the black ones are either literature-based approximations or intuitively chosen hypothetical values (kept constant among sites). During sensitivity analysis, init was halved.

| **Alga** | init | 500 |
| --- | --- | --- |
|  | death | 500 |
|  | birth | 500 |
|  | when | 5000 |
| **Carn** | init | 5 |
|  | death | 0.5 |
|  | birth | 50 |
| **Colf** | init | 212 |
|  | death | 0.214 |
|  | birth | 214000 |
| **Colg** | init | 385 |
|  | death | 0.385 |
|  | birth | 385000 |
| **Diat** | init | 500 |
|  | death | 500 |
|  | birth | 500 |
|  | when | 5000 |
| **Graz** | init | 1085 |
|  | death | 1.085 |
|  | birth | 108500 |
| **Hede** | init | 10 |
|  | death | 1 |
|  | birth | 1000 |
| **Herb** | init | 10 |
|  | death | 1 |
|  | birth | 1000 |
| **Leaf** | init | 1000 |
|  | death | 10000 |
|  | birth | 10000 |
|  | when | 100000 |
| **Omni** | init | 10 |
|  | death | 1 |
|  | birth | 100 |
| **POM** | init | 1000 |
|  | death | 10000 |
|  | birth | 10000 |
|  | when | 100000 |
| **Pred** | init | 63 |
|  | death | 6.3 |
|  | birth | 6300 |
| **Shre** | init | 87 |
|  | death | 0.114 |
|  | birth | 114000 |
| **Terr** | init | 54 |
|  | death | 5.4 |
|  | birth | 540 |
|  | when | 54 |

| i | j |  |
| --- | --- | --- |
| **Pred** | **Carn** | 0.111 |
| **Graz** | **Carn** | 0.111 |
| **Colf** | **Carn** | 0.111 |
| **Colg** | **Carn** | 0.111 |
| **Omni** | **Carn** | 0.111 |
| **Shre** | **Carn** | 0.111 |
| **Terr** | **Carn** | 0.111 |
| **Herb** | **Carn** | 0.111 |
| **Hede** | **Carn** | 0.111 |
| **Pred** | **Omni** | 0.111 |
| **Colg** | **Omni** | 0.111 |
| **Graz** | **Omni** | 0.111 |
| **Colf** | **Omni** | 0.111 |
| **Shre** | **Omni** | 0.111 |
| **Terr** | **Omni** | 0.111 |
| **Alga** | **Omni** | 0.111 |
| **POM** | **Omni** | 0.111 |
| **Diat** | **Omni** | 0.111 |
| **Graz** | **Pred** | 0.25 |
| **Colg** | **Pred** | 0.25 |
| **Colf** | **Pred** | 0.25 |
| **Shre** | **Pred** | 0.25 |
| **Diat** | **Hede** | 0.25 |
| **POM** | **Hede** | 0.25 |
| **Leaf** | **Hede** | 0.25 |
| **Alga** | **Hede** | 0.25 |
| **Diat** | **Herb** | 0.333 |
| **Alga** | **Herb** | 0.333 |
| **POM** | **Herb** | 0.333 |
| **Alga** | **Graz** | 0.07 |
| **Diat** | **Graz** | 0.196 |
| **POM** | **Graz** | 0.734 |
| **POM** | **Colg** | 1 |
| **POM** | **Colf** | 1 |
| **Leaf** | **Shre** | 1 |
