# Supplementary material for: Simulating Food Web Dynamics along a Gradient: Quantifying Human Influence
Source: PLoS One. 2012 Jul 2;7(7):e40280. doi: 10.1371/journal.pone.0040280 (PMC3388060; doi:10.1371/journal.pone.0040280)
Supplement: Appendix S3 — The food web at site 1, size of nodes being proportional to IH(M) and KH(M). (DOC) [file pone.0040280.s003.doc]

**Appendix S3:**

The food web of the pristine river (at site 1), the size of nodes is proportional to *IH(M)* (a) and *KH(M)* (b). Network drawn by COSBILAB Graph [17].

**
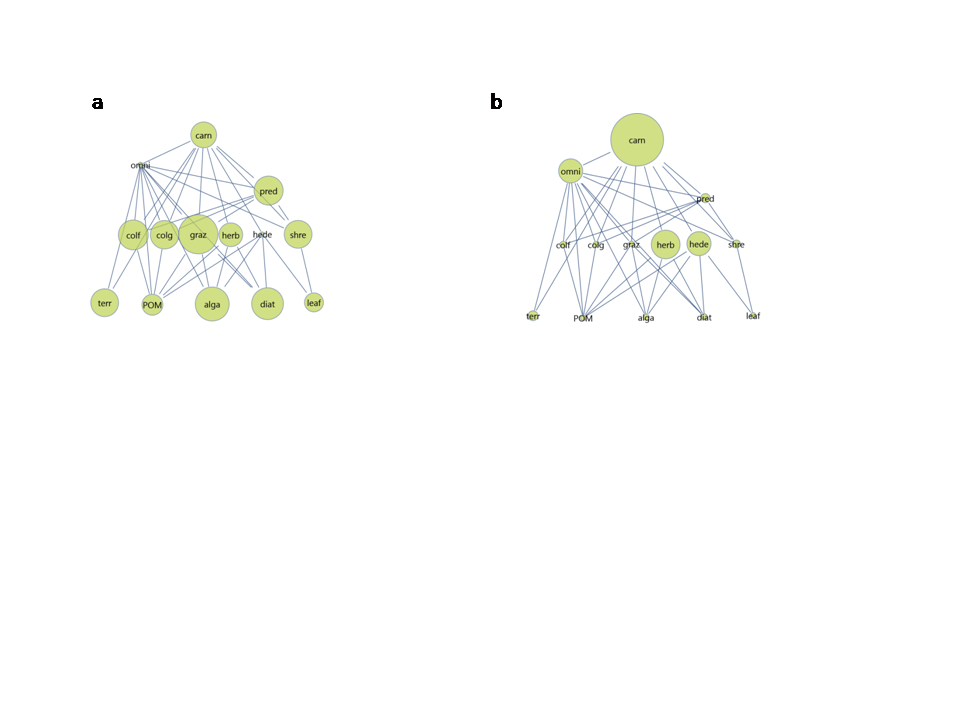
**
