# Supplementary figures and images for: Simulating Food Web Dynamics along a Gradient: Quantifying Human Influence
Source: PLoS One. 2012 Jul 2;7(7):e40280. doi: 10.1371/journal.pone.0040280 (PMC3388060; doi:10.1371/journal.pone.0040280)

**Appendix S4:**

The *IH(M)* (a) and *IH(V)* (b) values for each trophic group at each site.

**
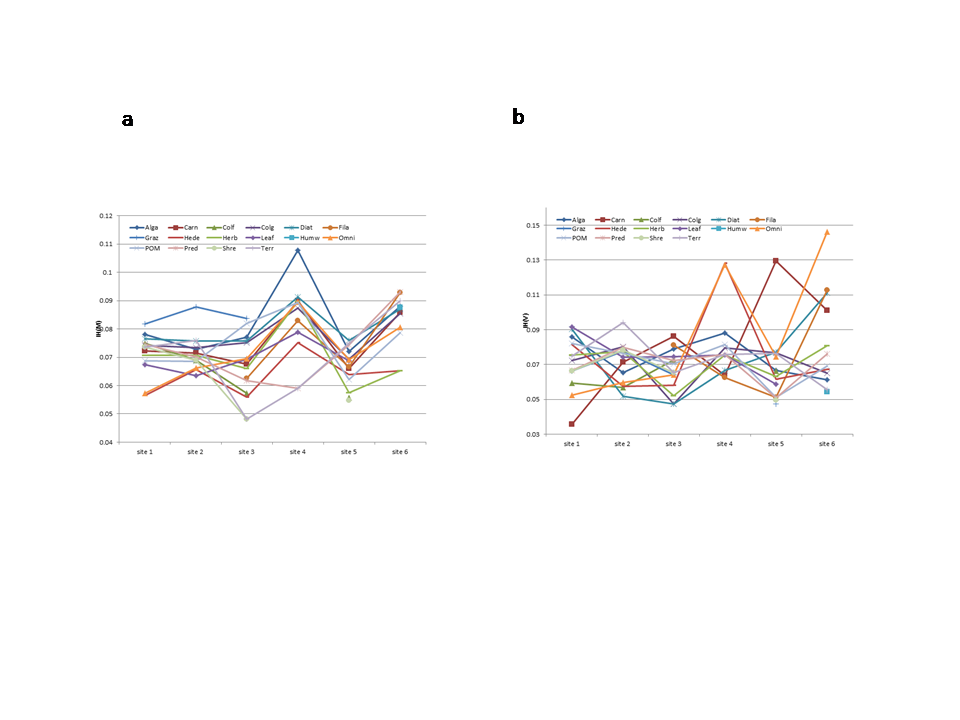
**

Supplement: Appendix S4 — The IH(M) and IH(V) values of trophic groups at the six sites. (DOC) [file pone.0040280.s004.doc]
