# Supplementary material for: Simulating Food Web Dynamics along a Gradient: Quantifying Human Influence
Source: PLoS One. 2012 Jul 2;7(7):e40280. doi: 10.1371/journal.pone.0040280 (PMC3388060; doi:10.1371/journal.pone.0040280)
Supplement: Appendix S5 — The importance rank of trophic groups based on IH(M) and IH(V). (DOC) [file pone.0040280.s005.doc]

**Appendix S5:**

The importance rank of trophic groups according to the *IH(M)* and *IH(V)*) indices in each

of the six sites.

|  | **site 1** |  | **site 2** |  | **site 3** |  | **site 4** |  | **site 5** |  | **site 6** |
| --- | --- | --- | --- | --- | --- | --- | --- | --- | --- | --- | --- |
|  | **I_H_(M)** |  | **I_H_(M)** |  | **I_H_(M)** |  | **I_H_(M)** |  | **I_H_(M)** |  | **I_H_(M)** |
| Graz | 0,0818 | Graz | 0,0878 | Graz | 0,0838 | Alga | 0,1078 | Diat | 0,0759 | Pred | 0,0931 |
| Alga | 0,0780 | Terr | 0,0759 | POM | 0,0820 | Diat | 0,0913 | Terr | 0,0749 | Fila | 0,0929 |
| Diat | 0,0765 | Diat | 0,0758 | Alga | 0,0771 | Carn | 0,0900 | Pred | 0,0744 | Terr | 0,0900 |
| Colf | 0,0751 | Colg | 0,0735 | Diat | 0,0757 | Herb | 0,0897 | Alga | 0,0720 | Alga | 0,0880 |
| Pred | 0,0745 | Alga | 0,0729 | Colg | 0,0751 | Omni | 0,0896 | Colg | 0,0693 | Humw | 0,0876 |
| Colg | 0,0741 | Carn | 0,0714 | Omni | 0,0696 | POM | 0,0893 | Omni | 0,0692 | Diat | 0,0871 |
| Terr | 0,0736 | Herb | 0,0707 | Leaf | 0,0694 | Colg | 0,0874 | Graz | 0,0688 | Carn | 0,0859 |
| Shre | 0,0736 | Pred | 0,0702 | Carn | 0,0676 | Fila | 0,0829 | Leaf | 0,0688 | Colg | 0,0854 |
| Carn | 0,0722 | Shre | 0,0690 | Herb | 0,0659 | Leaf | 0,0789 | Fila | 0,0666 | Omni | 0,0807 |
| Herb | 0,0707 | Colf | 0,0689 | Fila | 0,0624 | Hede | 0,0752 | Carn | 0,0660 | POM | 0,0787 |
| POM | 0,0687 | POM | 0,0686 | Pred | 0,0618 | Pred | 0,0590 | Hede | 0,0640 | Hede | 0,0653 |
| Leaf | 0,0673 | Omni | 0,0662 | Colf | 0,0574 | Terr | 0,0590 | POM | 0,0622 | Herb | 0,0653 |
| Omni | 0,0572 | Hede | 0,0656 | Hede | 0,0560 |  |  | Herb | 0,0574 |  |  |
| Hede | 0,0566 | Leaf | 0,0636 | Shre | 0,0481 |  |  | Colf | 0,0557 |  |  |
|  |  |  |  | Terr | 0,0481 |  |  | Shre | 0,0547 |  |  |
|  |  |  |  |  |  |  |  |  |  |  |  |
|  | **I_H_(V)** |  | **I_H_(V)** |  | **I_H_(V)** |  | **I_H_(V)** |  | **I_H_(V)** |  | **I_H_(V)** |
| Leaf | 0,0915 | Terr | 0,0940 | Carn | 0,0863 | Hede | 0,1283 | Carn | 0,1294 | Omni | 0,1463 |
| Diat | 0,0903 | Colg | 0,0801 | Fila | 0,0813 | Omni | 0,1271 | Diat | 0,0769 | Fila | 0,1126 |
| Alga | 0,0860 | Pred | 0,0800 | Alga | 0,0789 | Alga | 0,0879 | Colg | 0,0768 | Diat | 0,1110 |
| POM | 0,0821 | Shre | 0,0784 | Leaf | 0,0746 | POM | 0,0813 | Terr | 0,0763 | Carn | 0,1010 |
| Hede | 0,0813 | Graz | 0,0777 | Colf | 0,0726 | Colg | 0,0797 | Omni | 0,0744 | Herb | 0,0807 |
| Herb | 0,0755 | Herb | 0,0772 | Pred | 0,0724 | Pred | 0,0759 | Alga | 0,0666 | Pred | 0,0761 |
| Terr | 0,0748 | POM | 0,0763 | POM | 0,0702 | Terr | 0,0759 | Colf | 0,0664 | POM | 0,0690 |
| Colg | 0,0723 | Leaf | 0,0741 | Shre | 0,0654 | Herb | 0,0755 | Herb | 0,0632 | Hede | 0,0672 |
| Pred | 0,0666 | Carn | 0,0713 | Terr | 0,0654 | Leaf | 0,0754 | Hede | 0,0616 | Colg | 0,0650 |
| Shre | 0,0663 | Alga | 0,0653 | Omni | 0,0642 | Diat | 0,0666 | Leaf | 0,0586 | Alga | 0,0611 |
| Graz | 0,0661 | Omni | 0,0595 | Graz | 0,0642 | Carn | 0,0639 | POM | 0,0512 | Terr | 0,0556 |
| Colf | 0,0593 | Hede | 0,0575 | Hede | 0,0579 | Fila | 0,0625 | Fila | 0,0511 | Humw | 0,0543 |
| Omni | 0,0524 | Colf | 0,0570 | Herb | 0,0521 |  |  | Pred | 0,0509 |  |  |
| Carn | 0,0355 | Diat | 0,0516 | Diat | 0,0473 |  |  | Shre | 0,0496 |  |  |
|  |  |  |  | Colg | 0,0472 |  |  | Graz | 0,0472 |  |  |
